# Bayesian Space-Time Patterns and Climatic Determinants of Bovine Anaplasmosis

**DOI:** 10.1371/journal.pone.0151924

**Published:** 2016-03-22

**Authors:** Gregg A. Hanzlicek, Ram K. Raghavan, Roman R. Ganta, Gary A. Anderson

**Affiliations:** 1 Kansas State Veterinary Diagnostic Laboratory and Department of Diagnostic Medicine/Pathobiology, College of Veterinary Medicine, Kansas State University, Manhattan, Kansas, United States of America; 2 Center of Excellence for Vector-Borne Diseases, Department of Diagnostic Medicine/Pathobiology, College of Veterinary Medicine, Kansas State University, Manhattan, Kansas, United States of America; University of Minnesota, UNITED STATES

## Abstract

The space-time pattern and environmental drivers (land cover, climate) of bovine anaplasmosis in the Midwestern state of Kansas was retrospectively evaluated using Bayesian hierarchical spatio-temporal models and publicly available, remotely-sensed environmental covariate information. Cases of bovine anaplasmosis positively diagnosed at Kansas State Veterinary Diagnostic Laboratory (*n* = 478) between years 2005–2013 were used to construct the models, which included random effects for space, time and space-time interaction effects with defined priors, and fixed-effect covariates selected *a priori* using an univariate screening procedure. The Bayesian posterior median and 95% credible intervals for the space-time interaction term in the best-fitting covariate model indicated a steady progression of bovine anaplasmosis over time and geographic area in the state. Posterior median estimates and 95% credible intervals derived for covariates in the final covariate model indicated land surface temperature (minimum), relative humidity and diurnal temperature range to be important risk factors for bovine anaplasmosis in the study. The model performance measured using the Area Under the Curve (AUC) value indicated a good performance for the covariate model (> 0.7). The relevance of climatological factors for bovine anaplasmosis is discussed.

## Introduction

Bovine anaplasmosis, caused by an obligate intercellular bacterium, *Anaplasma marginale* (Family: Anaplasmataceae, Order: Rickettsiales) affects beef-cattle and dairy production in almost all the states in the US, causing significant economic losses to producers. The control of this disease currently relies predominantly on infection-avoidance alone since fully licensed vaccines are not marketed in North America (*see also*, http://www.anaplasmosisvaccine.com/). The bacterium is known to cause a hemolytic disease in cattle, which manifests as anemia, abortion, icterus, lethargy, and causes death primarily in older animals [1]. Cattle that survive infection are persistent carriers of the pathogen and are a source of infection for other animals through inadvertent mechanical transmission via blood-contaminated multi-use needles and surgical equipment and as well as via tick transmission. In North America, *A*. *marginale* is biologically vectored by different hard tick species in the Genus *Dermacentor* [2], [3] but other arthropod vectors that could aid in the transmission of this disease may also include ticks in the Rhipicephalus Genus and biting flies [3].

Illnesses caused by tick-borne pathogens to animals and as well as humans in general have increased over the past years in the Midwestern US [4] [5], including in the state of Kansas where tick-borne diseases have expanded to newer areas over the years that have traditionally not witnessed such diseases in the past [6], [7]. Some of the increase in the space-time expansion of tick-borne diseases in the Midwestern region may be attributed to geographic expansion of tick populations [8–10]. Although a plethora of anecdotal and published evidence suggest the increasing menace of bovine anaplasmosis in newer areas, quantitative space-time evaluation of whether or not bovine anaplasmosis has spread to previously unreported areas over time is not readily available. Likewise, information on any potential environmental and climatological drivers behind the space-time expansion of bovine anaplasmosis cannot be easily found, which has disease management implications.

Space-time disease mapping models are a popular tool to describe disease patterns and to identify unusual clusters of incidence in space and time-trends or both. Bayesian hierarchical models with different parametric or non-parametric time-trend and space-time interactions have advantages over frequentist approaches for analyzing datasets with inherent space-time dependency [11], [12]. Such models help detect any localized clusters that may be linked in time, for instance due to a set of favorable environmental drivers or cattle movement. Another way to strengthen inference from Bayesian space-time models is by including relevant ecological covariates that often explain additional variability in aggregated incidence datasets. This is particularly relevant in the case of tick-borne disease incidences since the spatial distribution of ticks are largely determined by physical environment and climatological conditions.

The objective of the study reported here was to retrospectively evaluate the space-time patterns and the environmental drivers of bovine anaplasmosis incidence in the Midwestern state of Kansas using Bayesian hierarchical modeling approach. Cases used in the study were diagnosed at Kansas State Veterinary Diagnostic Laboratory (KSVDL) between the years 2005–2013.

## Materials and Methods

### 2.1. Anaplasmosis data

Positive test records for bovine anaplasmosis were searched through the Laboratory Information Management System (LIMS) at Kansas State Veterinary Diagnostic Laboratory (KSVDL), and summarized to their respective counties. Diagnostic test results that indicated a positive diagnosis for anaplasmosis in one or more of the following tests, including blood smear, ELISA, or polymerase chain reaction test were considered as confirmed cases of bovine anaplasmosis.

### 2.2. Covariate data

Covariates representing the physical environment were derived from the National Land Cover Dataset (NLCD) [13], and climatological data were derived from USGS and NASA resources (Table 1). The environmental covariates represented percentages of different land classes for each county in the state of Kansas, and as well as two variables representing landscape metrics indicative of landscape fragmentation viz., total edge contrast index and patch density. The total edge contrast index was calculated in FRAGSTATS [14] program by

$$TECI={\left[\sum_{i=1}^{m}\sum_{k=i+1}^{m}e_{ik}d_{ik}\right]}^{-E^{*}}(100).$$ (1)

where *e*_*ik*_ is the total length of edge between patch types *i* and *k*, and E* is the total length of edge in landscape, and *d*_*ik*_ is the dissimilarity (edge contrast weight) between patches *i* and *k*. Patch density was estimated in FRAGSTATS program by

$$PD=\frac{N}{A}(10,000)\;(100).$$ (2)

where *N* = total number of patches in the landscape and *A* = total landscape area (*m*^2^).

**Table 1:** Physical environment and climatological covariates evaluated in the study.

Variable	Mean	S.D	Minimum	Maximum	*P-value*
^£^*Land cover land use and landscape metrics*:					
Open water	0.94	0.88	0.18	2.13	0.38
Developed–open space	1.68	0.89	0.91	4.51	0.47
Barren land	0.01	0.01	0.00	0.10	0.68
Deciduous forest	3.28	3.10	1.91	4.20	0.30
Evergreen forest	4.24	5.75	1.81	4.71	0.28
Mixed forest	1.71	0.91	0.57	2.10	0.71
Scrub/shrub	2.54	1.98	1.85	3.51	0.55
Grassland/herbaceous	52.5	14.2	39.1	62.2	0.15
Pasture/hay	0.81	0.79	0.47	1.32	0.24
Cultivated crops	29.12	26.8	18.9	39.4	0.22
Woody wetlands	0.64	0.61	0.24	1.21	0.81
Emergent herbaceous wetland	0.04	0.03	0.00	0.08	0.41
Total edge contrast index	43.2	21.5	27.12	51.27	0.19
Patch density	57.6	53.1	31.51	61.21	0.64
*Climate*:					
^¶^Maximum Normalized Vegetation Index (NDVI)	0.35	0.31	0.31	0.40	0.28
Minimum Land Surface Temperature	16.8	8.5	14.21	24.10	0.03
Mean Land Surface Temperature)	29.1	18.2	27.21	32.17	0.30
Diurnal Temperature Range (DTR) ^±^	24.51	11.25	21.71	29.61	0.04
Precipitation,	27.12	21.24	13.57	34.25	0.23
Humidity	57.21	24.11	39.24	78.25	0.01

^£^ Source: MRLC (2011); years^1^: 2001–2011; resolution: 30 m; spatial scale^3^: 1:100,000).

^¶^ Source: NDVI, Minimum and Mean Land Surface Temperature were obtained from MODIS (Moderate Resolution Imaging Spectroradiometer), LP DAAC; and Diurnal Temperature Range, precipitation, humidity were obtained from (POWER, NASA Langley Research Center).

^±^ The difference between daily maximum and minimum temperatures averaged over a thirty day period).

Climatic variables including the maximum normalized vegetation index (NDVI), minimum land surface temperature *LST*_(min)_, mean *LST*_(*mean*)_, diurnal temperature range (DTR) (the difference between daily maximum and minimum temperatures averaged over a thirty day period), precipitation and humidity were extracted for each county in the study area. The *LST* and NDVI estimates were derived from MODIS (Moderate Resolution Imaging Spectroradiometer) imagery [15]. DTR, precipitation and relative humidity were derived from the Prediction of Worldwide Renewable Energy (POWER) web portal of the NASA Langley Research Center [16] [17]. All climatological data were downloaded for a period roughly corresponding to high tick activity season in the region (March through August) and averaged to derive representative values.

To account for reservoir host effect in the models, the number of deer per Deer Management Unit (DMU) was obtained for the study region from the Kansas Department of Wildlife, Parks and Tourism (KDWPT). Since the DMUs were at a coarser scale than counties, deer numbers for all the counties that were completely present within a given DMU and those whose 50% or more land area was present within a DMU was assigned the same value, then divided by the area of the respective counties to obtain density values.

### 2.3. Statistical analyses and model specification

Let *Y*_*ij*_ be the observed number of infected cattle among *N*_*ij*_ individual cattle at risk within a population in county *i*, diagnosed positive in year *j*. We modelled *Y*_*ij*_ to follow a binomial approximation *Y*_*ij*_ ∼ *bin*(*θ*_*ij*_), where *θ*_*ij*_ is the expected number of cattle that are at risk for anaplasmosis in county *i* in year *j*. The probability of detecting anaplasmosis *θ*_*ij*_ is given by

$$\left(\theta_{ij})=\sum_{k=1}^{\Pi }(X_{ij}^{\left(k\right)}\right)$$ (3)

where, $X_{ij}={\left(X_{ij}^{\left(1\right)},X_{ij}^{\left(2\right)},\ldots ,X_{ij}^{\left(\Pi \right)}\right)}^{T}$ is the vector of Π associated with environmental predictors *k* observed at county *i*.

Univariate regression models were run to identify physical environmental and climatic factors significantly associated with anaplasmosis risk. Candidate explanatory variables to be included in the Bayesian hierarchical models were screened *a priori* in order to avoid model fitting issues. Several Frequentist bivariate regression models evaluated each variable independently and variables that were significant at *p* < 0.2 were kept. Care was taken not to remove candidate variables that were deemed clinically relevant. Multicollinearity among screened variables was tested by estimating the variance inflation factor (*VIF*) and all variables with a *VIF* ≥ 10 were considered to indicate multicollinearity, in which case, one of the variables was dropped at a time until multicollinearity was absent. Non-linearity among independent variables with the response was evaluated at the screening stage with univariate regressions, and when non-linear variables were present, they were categorized using cutoffs based on scatter-plots.

Bayesian geostatistical models with county-specific random effects were fitted to estimate the degree of spatial autocorrelation in anaplasmosis risk and to assess the effect of different covariates. For the process models, we used a logit link function in an extended generalized linear model (*GLM*) structure that incorporated stochastic spatial and temporal functions and as well as different covariate effects. Several models that allowed us to evaluate random and covariate effects on anaplasmosis prevalence were fitted individually. First, we modelled the spatial component we adopted the standard Besag et al (1991) [18] model with a spatially unstructured and structured *u*_*i*_,*v*_*i*_ components.

$$\left(\theta_{ij})=\beta_{0}+u_{i}+v_{i}.\;(\mathrm{model}-1\right)$$ (4)

Where, *β*_0_ (intercept) represents the mean prevalence of anaplasmosis in all counties in all years, and *u*_*i*_,*v*_*i*_ are a random terms accounting for spatially unstructured variation in anaplasmosis prevalence and unstructured heterogeneity, respectively. No interaction was assumed to exist between *u*_*i*_ and *v*_*i*_ were assigned *u*_*i*_ ∼ *CAR*, $v_{i}\sim Normal(0,\sigma_{v}^{2})$ priors. Spatial dependence in *u*_*i*_ was applied by assuming a conditional autoregressive model (*CAR*)(*γ*) with a Gaussian distribution [19], which implies that each *u*_*i*_ is conditional on the neighbor *u*_*j*_ with variance $\sigma_{i}^{2}$ dependent on the number of neighboring counties *n*_*i*_ of county *i*, i.e.,

$$u_{i}|u,j\;neighbor\;of\;i∼N\left(\frac{1}{n_{i}}\gamma \sum_{j=1}^{n_{i}}u_{j},\frac{\sigma_{i}^{2}}{n_{i}}\right)$$ (5)

In a second model we introduced a *γ*_*j*_ term to account for the temporal component of the data. This term was assigned a random walk (RW1) prior $\gamma_{j}\sim N(\gamma_{j-1},\tau_{\gamma }^{-1}).$ [20]

$$\left(\theta_{ij})=\beta_{0}+u_{i}+v_{i}+\gamma_{j}.\;(\text{model-}2\right)$$ (6)

Following this in a third model, in order to detect potential space-time interaction effects in anaplasmosis prevalence, a random term *ψ*_*ij*_ was introduced, with *ψ*_*ij*_ ∼ *N*(*ψ*_*i*,*j*−1_*τ*_*ψ*_) prior [21]. The model was notated as,

$$\left(\theta_{ij})=\beta_{0}+u_{i}+v_{i}+\psi_{ij}.\;(\text{model-}3\right)$$ (7)

For the covariate model, different covariates were included to space-time model in several steps, starting with a model that included all covariates followed by removal of one covariate at each step. Covariates were retained in the model unless their removal resulted in the increase of Deviance Information Criterion (DIC) [22] value by 5 units or more. Interaction effects between covariates were included in these steps as well, previously removed covariates did not enter the final model.

The model posterior parameters were estimated using a Bayesian framework implemented using R-INLA software on a Linux Beocat cluster computing environment. Distributions of covariate effects on anaplasmosis prevalence are seldom available for the region; therefore non-informative, uniform priors were selected for the regression parameters, *β*_*k*_ and their variance components, $\sigma_{k}^{2}.$ This allowed the observed data to have the greatest influence on posterior distributions without being constrained by the choice of a prior. The median estimates from the posterior Bayes distribution and their 95% Credible Intervals (*CrI*) were calculated.

Models were validated by randomly partitioning the county-level expected risk estimates into five subsets and by running the models using only four of the five subsets, while validating model prediction with the fifth subset. The models were run for five times to allow each validation with subset. Each time, the model’s performance (prediction accuracy) was measured using area under the receiver-operator’s curve (*AUC*) [23] values with the observed prevalence (dichotomized as 0,≥ 0). The mean error and mean absolute error were calculated to quantify prediction bias and overall precision respectively.

## Results

**Fig 1:**
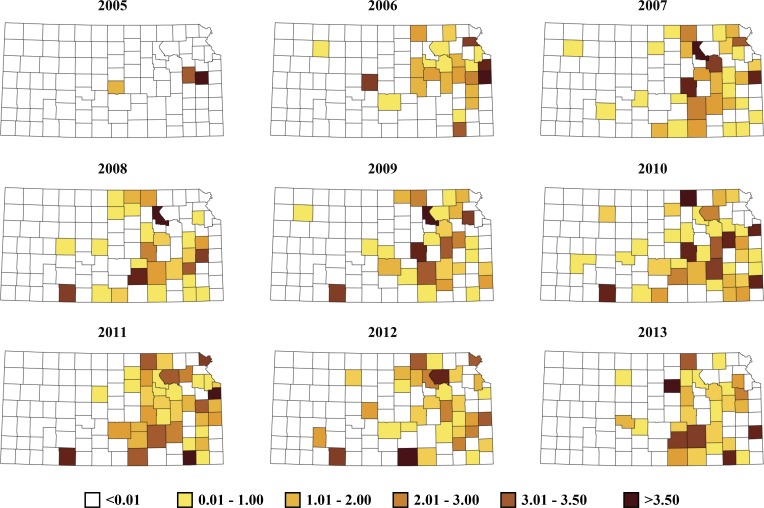
County-level crude rate ratios (observed) for positive bovine anaplasmosis test results conducted at Kansas State Veterinary Diagnostic Laboratory (KSVDL) between years 2005–2013.

There were *n* = 478 cases of bovine anaplasmosis diagnosed positive at KSVDL between years 2005–2013, which were distributed predominantly in counties to the east central and some western parts in Kansas (Fig 1). There was a large variability in the average annual number of cases aggregated at the respective counties. No tests were submitted from many counties throughout the study period (Fig 1), and for others the annual averages ranged from 4 to 12 between 2005–2013.

Of the three progressively additive partial space-time models evaluated in the study, model-3 had the lowest DIC value, indicating the presence of a time trend and space-time interaction effect in the bovine anaplasmosis dataset. In the ensuing steps, different covariates were added to this model (model-3) alone. Of all the covariates evaluated in the study, six retained significance in the univariate screening with a liberal *p* ≤ 0.2 level (Table 2). Multicollinearity and nonlinearity among the six selected candidate variables were absent.

**Table 2:** Results of univariate regression analysis and candidate variables selected for Bayesian analysis (*p* ≤ 0.2).

Covariate	Estimates	S.E	*p–value*
Total edge contrast index	-2.04	1.27	0.19
% grassland vegetation	1.21	0.97	0.15
Minimum Land Surface Temperature	0.93	0.11	0.03
Relative humidity	1.84	0.12	0.01
Diurnal temperature range	1.05	0.08	0.04
Deer density	0.89	0.31	0.15

The partial space-time model with lowest *DIC* value (model-3) and the best fitting covariate model (model-7) and had similar *DIC* values (Table 3); although, the covariate model had the lowest *DIC* value among all models and all interpretations were made based on this model alone. The best fitting covariate model indicated a significant spatiotemporal effect for all years, and three climatological covariates, minimum land surface temperature (henceforth *LST*_(min)_), relative humidity and diurnal temperature range (henceforth DTR) were retained as significant determinants of bovine anaplasmosis in the study region (Table 4). The odds ratios and 95% *CrI* for different covariates in the models are present in Table 4, and they indicate that those counties with higher annual averages of *LST*_(min)_, relative humidity and diurnal temperature significantly increased the odds of positive diagnosis for anaplasmosis in cattle. No physical environment variable were retained in the final covariate model.

**Table 3:** Model fit and comparison criteria.

Model	$\bar{D}$	*p*_*D*_	*DIC*
*Partial space-time model*			
1	7094	325	7419
2	5420	421	5841
3	3354	251	3605
*Covariate model*			
4	5229	514	5743
5	4400	751	5151
6	3864	548	4412
7	3095	421	3516

**Table 4:** Model statistics for Bayesian spatio-temporal covariate models evaluating county-level bovine anaplasmosis prevalence in Kansas, USA. Posterior median Bayes estimates (95% Bayesian credible intervals).

Covariate	Model 4	Model 5	Model 6	Model 7
Total edge contrast index	-1.76 (-3.13, 1.01)	-	-	-
% grassland vegetation	0.68 (0.07, 1.05)	0.61 (0.07, 1.25)	-	-
Minimum land surface temperature	0.87 (0.10, 0.91)	0.89 (0.12, 0.91)	0.87 (0.10, 0.91)	0.87 (0.10, 0.91)
Relative humidity	1.51 (1.11, 2.14)	1.51 (1.12, 2.14)	1.53 (1.14, 2.14)	1.53 (1.15, 2.14)
Diurnal temperature range	0.77 (0.21, 0.84)	0.77 (0.21, 0.83)	0.77 (0.21, 0.82)	0.77 (0.22, 0.81)
Deer density	0.54 (0.47, 0.97)	0.54 (0.52, 0.98)	0.54 (0.52, 0.99)	-

**Fig 2:**
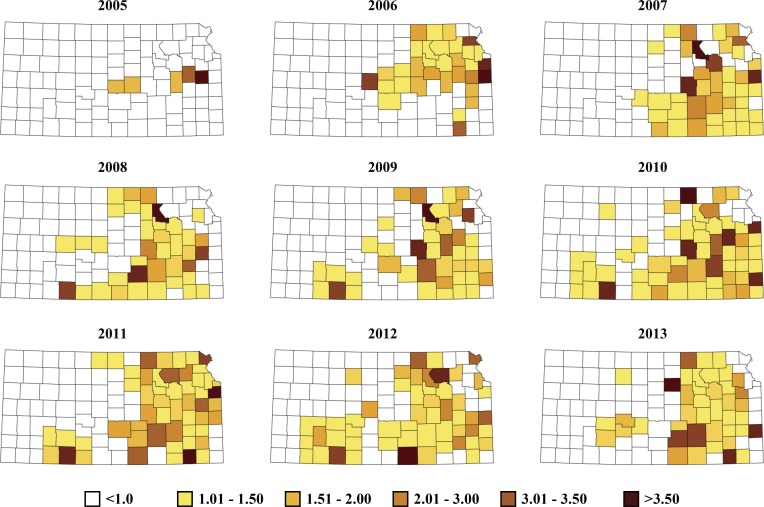
Smoothed county-level expected risk maps for bovine anaplasmosis in Kansas from 2005–2013.

**Fig 3:**
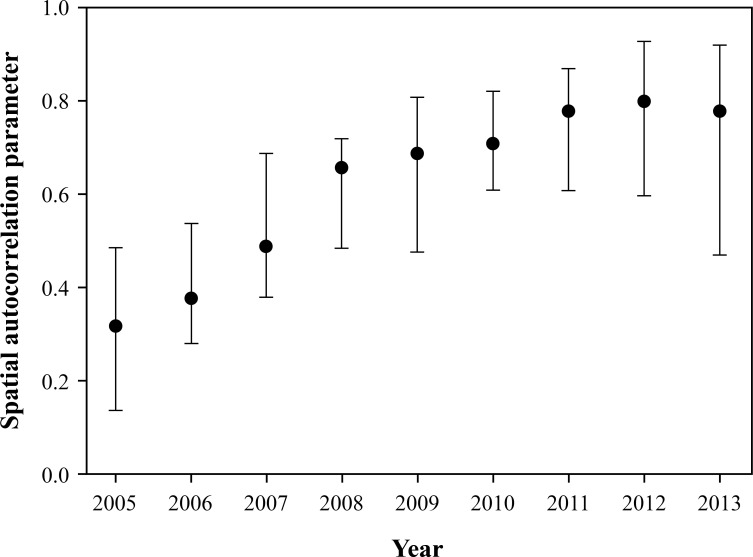
Spatial autocorrelation parameter (posterior estimates) and 95% CrI for county level bovine anaplasmosis expected risk between years 2005–2013.

The observed county-level aggregates of positive bovine anaplasmosis cases per county, per year is present in Fig 1, and the posterior estimates based on the final covariate model, which also included random terms for space, time and space-time effects is present in Fig 2. The space-time parameter estimates and their 95% credible intervals throughout the study period indicated a steady progression of bovine anaplasmosis for the study region over the years with the exception of the years following 2011, and a slight decline for years 2012 and 2013 (Fig 3). However, the trend observed for the later part of the study period may be an artifact of the inability of the covariate model to capture all the variability in the dataset for these years and not an actual decline in the space-time trend.

A comparison of model precision measured by the area under receiver-operator curve (*AUC*) for the partial space-time model (model-3) and the covariate model (model-7) is present in Table 5, which indicated a good discriminative capacity for the covariate model, and the mean error and mean absolute error indicated that covariate model performed relatively better than other models considered (Table 5).

**Table 5:** Model validation summary for bovine anaplasmosis prevalence in Kansas between years 2005–2013 predicted by a partial Bayesian space-time model (model–3) and the final covariate model (model–7).

Year	Model	AUC	Mean error	Mean absolute error
2005				
	Partial	0.61	0.12	7.22
	Covariate	0.70	-0.18	6.18
2006				
	Partial	0.58	0.24	7.05
	Covariate	0.70	0.08	5.81
2007				
	Partial	0.66	0.18	6.25
	Covariate	0.72	0.11	4.28
2008				
	Partial	0.66	0.15	6.01
	Covariate	0.74	0.14	3.81
2009				
	Partial	0.62	0.16	6.05
	Covariate	0.75	0.14	3.57
2010				
	Partial	0.70	0.12	5.83
	Covariate	0.77	0.11	3.48
2011				
	Partial	0.69	0.13	4.81
	Covariate	0.74	0.13	4.22
2012				
	Partial	0.65	0.15	5.61
	Covariate	0.71	0.10	4.01
2013				
	Partial	0.66	0.15	6.05
	Covariate	0.71	0.08	4.44

**Fig 4:**
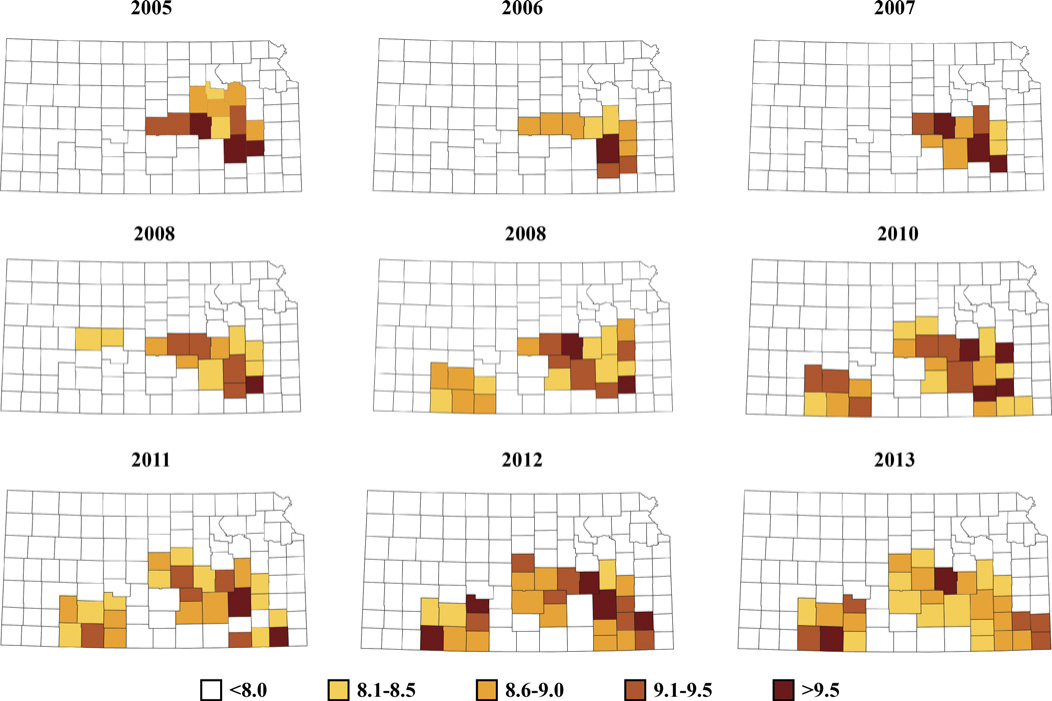
Plot of space-time interaction effect (*ψ*_*ij*_) for bovine anaplasmosis incidence in Kansas between years 2005–2013.

To highlight the counties having the most substantial space-time interactions, we plotted the posterior probability of *ψ*_*ij*_ > 1 is above 0.8, and the results are plotted in Fig 4. The interaction terms are predominant in the central and south eastern counties of Kansas, indicating that common risk factors are coming to play in these areas for bovine anaplasmosis.

## Discussion

This study has identified a significant space-time progression of bovine anaplasmosis in the central Midwestern state of Kansas along with important climatological drivers of the space-time progression. The posterior Bayesian estimates derived in this study for the covariate model were based on individual numbers of tests that indicated positive diagnosis for bovine anaplasmosis cases submitted to KSVDL. However, tests for anaplasmosis are often referred to laboratories based on blood drawn from small groups of cattle within a herd, and it is highly likely for other animals in the herd to be infected as well. Using infected herd numbers per county, per year instead of individual tests on cattle numbers would yield more accurate posterior estimates. Despite our best attempts, data pertaining to herd sizes and the number of herds per county could not be obtained for this study. Notwithstanding this limitation the covariate model performed satisfactorily in predicting infection occurrences as indicated by the model performance statistics (Table 4), and indicate a steady spatio-temporal progression of bovine anaplasmosis in the state of Kansas over the past several years (Fig 3).

A plot of counties with high space-time interaction in the covariate model consistently indicated the presence of a cluster of counties in the central and south central portion of the state (Fig 4), suggestive of common risk factors in this area for cattle infection with bovine anaplasmosis. The cluster of counties identified in the present study comprises an area in Kansas that is known for its higher tick spp. density; however, the prevalence of *A*. *marginale* among *Dermacentor* ticks or other potential tick species from this area is not clearly known. Studies to quantify prevalence levels of the bacterium in the cluster of counties identified here may yield useful management strategies. In addition to the role of ticks, the cluster identified here may also indicate the routine import of infected cattle from other states into these counties, and as well as prevalent management practices that could inadvertently sustain transmission of *A*. *margniale* to naïve cattle herds. Future epidemiological studies on bovine anaplasmosis in the state may benefit by considering these factors.

Widening spatial distribution of bovine anaplasmosis and the potential for increased intensity of this disease due to ongoing climate-change has been speculated in the past (e.g., Kocan et al., 2010, Jonsson and Reid, 2000) [3], [24]. The identification of climatological factors as important drivers of the noted space-time progression and as risk factors for bovine anaplasmosis in this study further supports this suggestion. Climatic conditions considered typical for the Central Plains region in the US have already been noted to have changed in noticeable ways due to climate-change [25], [26], and many such conditions are known to affect tick phenology and their spatial distribution either directly or indirectly. The identification of associations between climate-change indices and infectious diseases is often the first step in understanding climate-change impacts on infectious diseases. However, detecting such associations are often problematic, with some of the contributing factors to this problem being presence of multiple influential but also confounding factors for infectious diseases, variability in infection data due to host related factors, coarse resolution of disease and climate data used for detection of such associations. Climate covariates were more important than the physical environment in the present study, and all three climatological covariates, minimum land surface temperature, relative humidity and diurnal temperature range (the difference between daily minimum and maximum temperatures) are considered as indices of climate-change and are intensely monitored by the climate-change research community [27], [28].

Land surface temperature and diurnal temperature are relevant to tick survival, spatial distribution and potentially also their ability to successfully harbor and transmit pathogens. The *LST*_min_ identified in the study likely indicates the average minimum temperature in some counties as a limiting factor for ticks to survive through winters but not in others. The diurnal temperature range indicates the warming night time temperatures that may favor tick survival in their current ecological niche and as well as expansion to newer areas where conditions are becoming suitable. Diurnal temperature range has been decreasing worldwide since the 1950s due to increasing daily minimum temperature (*T*_min_) at a faster rate than the daily maximum temperature (*T*_max_), as well as (*T*_min_) decreasing at a slower rate than (*T*_max_). For most parts of the US, trends show that (*T*_max_) have remained constant or have increased only slightly but (*T*_min_) have increased at a faster rate [27] [29].

Relative humidity has been associated with the prevalence and distribution of other tick-borne diseases in North America [30–32] and it is an important delimiter to the survival and spatial distribution of ticks [33] [34]. There are large variations in the yearly precipitation received across the state of Kansas, with eastern Kansas receiving up to three times more rainfall than west [35]. As a result, climate and vegetation are transitional between the humid east and semi-arid western portion of Kansas that may explain the noted geographic pattern for bovine anaplasmosis in the present study. Humidity can often be seen associated with the survival and abundance of ticks in the literature, with higher humidity conditions often favoring the long-term survival of some ticks species’ life stages through dry seasons [36], [37] among other reasons. Of the three climatological covariates, relative humidity had the largest influence on the covariate model based on the posterior estimate and credible intervals. We are tempted therefore to suggest that the relatively higher relative humidity conditions in the eastern and southeastern parts of the state that favor the survival and proliferation of *A*. *marginale* transmitting tick vectors is by far the most important eco-climatic driver for bovine anaplasmosis in the region. Further field based and laboratory studies are essential to further strengthen this association.

## Conclusion

The study results indicate that cases of bovine anaplasmosis in Kansas has steadily increased between years 2005–2013 and to newer geographic areas during the same period. The presence of higher space-time interaction for bovine anaplasmosis infection within a cluster of central and south central counties indicate the influence of similar risk factors, and are potential areas for targeting prevention/management efforts. Three climate change indices, minimum land surface temperature, diurnal temperature range and relative humidity are drivers of space-time pattern for bovine anaplasmosis in Kansas at a county-scale. This finding is significant in a climate-change implications on infectious diseases context. Two immediate questions we are led to ask based on this finding are how the associations of these factors might be further quantified under field and laboratory conditions for the tick host and the pathogen? And, how these factors might influence the further geographic expansion of *Dermacentor* ticks and *A*. *marginale* under different climate-change scenarios.
